# Phylogenomics of non-model ciliates based on transcriptomic analyses

**DOI:** 10.1007/s13238-015-0147-3

**Published:** 2015-04-02

**Authors:** Xiao Chen, Xiaolu Zhao, Xiaohui Liu, Alan Warren, Fangqing Zhao, Miao Miao

**Affiliations:** 1 College of Life Sciences, University of Chinese Academy of Sciences, Beijing, 100049 China; 2 Institute of Evolution & Marine Biodiversity, Ocean University of China, Qingdao, 266003 China; 3 Department of Life Sciences, Natural History Museum, Cromwell Road, London, SW7 5BD UK; 4 Beijing Institutes of Life Science, Chinese Academy of Sciences, Beijing, 100101 China

**Keywords:** *Strombidium*, protozoa, transcriptome, non-model ciliate, phylogenomic analysis

## Abstract

Ciliates are one of the oldest living eukaryotic unicellular organisms, widely distributed in the waters around the world. As a typical marine oligotrich ciliate, *Strombidium sulcatum* plays an important role in marine food webs and energy flow. Here we report the first deep sequencing and analyses of RNA-Seq data from *Strombidium sulcatum*. We generated 42,640 unigenes with an N50 of 1,451 bp after *de* *novo* assembly and removing rRNA, mitochondrial and bacteria contaminants. We employed SPOCS to detect orthologs from *S. sulcatum* and 17 other ciliates, and then carried out the phylogenomic reconstruction using 127 single copy orthologs. In phylogenomic analyses, concatenated trees have similar topological structures with concordance tree on the class level. Together with phylogenetic networks analysis, it aroused more doubts about the placement of *Protocruzia*, *Mesodinium* and *Myrionecta*. While epiplasmic proteins are known to be related to morphological characteristics, we found the potential relationship between gene expression of epiplasmic proteins and morphological characteristics. This work supports the use of high throughput approaches for phylogenomic analysis as well as correlation analysis between expression level of target genes and morphological characteristics.

**Electronic supplementary material:**

The online version of this article (doi:10.1007/s13238-015-0147-3) contains supplementary material, which is available to authorized users.

## INTRODUCTION

Ciliates are unique among unicellular organisms in having two types of nuclei thus separating the germline and somatic functions and are therefore commonly used to study genome structure and gene expression (Aury et al., 2006). Over the last ten years, high throughput sequencing has made ciliate genomic research available on a large scale, and the genomes of several species have been sequenced and analyzed, e.g. *Tetrahymena thermophila* (Eisen et al., 2006), *Paramecium tetraurelia* (Auryet al., 2006), *Ichthyophthirius multifiliis* (Coyne et al., 2011) and *Oxytricha trifallax* (Swart et al., 2013). In addition, the transcriptomes of 13 ciliate species representing 10 genera and five classes are now available (Table S1) including those of *Tetrahymena thermophila* (Xiong et al., 2012) and *Chilodonella uncinata* (Grant et al., 2012).

**Figure 1:**
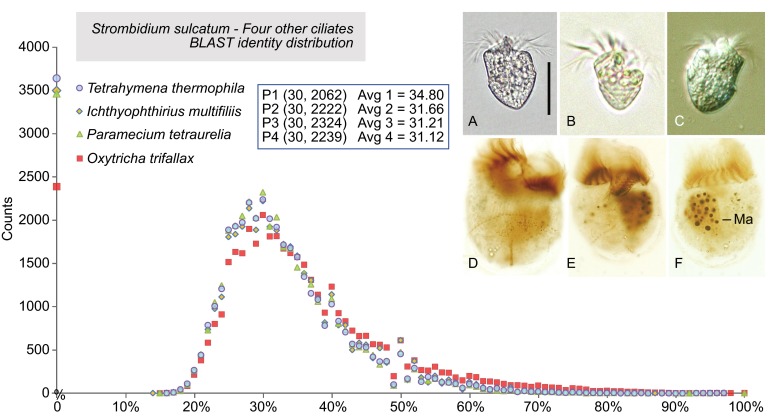
**BLAST identity distribution between proteins of** ***Strombidium sulcatum*** **and that of four other ciliates (** ***Oxytricha trifallax***, ***Tetrahymena thermophila***, ***Ichthyophthirius multifiliis***, ***Paramecium tetraurelia*** **)**. P(x,y), peaks (identity, peak value). Avg, average value. Photomicrographs of *Strombidium sulcatum* *in vivo* (A–C) and protargol-stained specimen (D–F). Ma, macronucleus. Scale bars are 25 µm

*Strombidium sulcatum* Claparède & Lachmann, 1859 (Fig. 1 A–F) is an oligotrich ciliate that plays an important role in food webs and energy flow in marine pelagic waters (Bernard & Rassoulzadegan, 1990, Montagnes et al., 1990). Since Claparède & Lachmann described *S. sulcatum* as the type species for the genus *Strombidium*, there have been numerous reports on its trophic status and diversity (Wiadnyana & Rassoulzadegan, 1989, e.g. Bernard & Rassoulzadegan, 1990, Allali et al., 1994, Christaki et al., 1998, Dolan et al., 2003). With the development of molecular sequencing technology, the taxonomic status of *S. sulcatum* and other species of *Strombidium* have been reassessed (Modeo et al., 2003, McManus et al., 2010, Li et al., 2013).

Ciliates are characterized by having basal bodies and associated appendages bounded to a submembrane cytoskeleton, the epiplasm, that lies beneath the inner alveolar membrane (Fauré-Fremiet, 1962). Two distinct classes of cytoskeletal protein have been identified in ciliates: articulins and epiplasmins (Nahon et al., 1993, Huttenlauch et al., 1995, Huttenlauch et al., 1998, Huttenlauch & Stick, 2003). These are homologous in protists and the presence of both has been established in the excavate flagellate *Euglena,* the dinoflagellate *Amphidinium* and the ciliates *Pseudomicrothorax, Paramecium, Tetrahymena* and *Euplotes* (Huttenlauch et al., 1998). Several novel families of articulins and epiplasmins have been identified including: plateins, which are articulin-related proteins in the ciliate *Euplotes* (Kloetzel et al., 2003a, b); EpiA, EpiB and EpiC, which are major cytoskeletal proteins in the cortex of *Tetrahymena* (Honts & Williams, 2003), and likewise Epi1-Epi51 in *Paramecium* (Pomel et al., 2006, Damaj et al., 2009, Aubusson-Fleury et al., 2013); alveolins, which are used to define the protist infrakingdom Alveolata (Gould et al., 2008). Most importantly, it had been shown that EpiC (Epc1p, gene: *EPC1*) influences both cell shape and the fidelity of cortical development (Huttenlauch et al., 1998).

Here we present Illumina Hiseq 2000 RNA-Seq data from *Strombidium sulcatum*. The transcriptome data are analyzed, including assembly and annotation. Phylogenomic reconstruction is carried out using 127 single copy orthologs, and evolutionary relationships are revealed by concatenated and concordance tree analyses. Thereafter, we investigate the transcriptional expression of epiplasmic proteins in *S. sulcatum* to determine whether there is a relationship between morphological characteristics and gene expression of epiplasmic proteins.

## RESULTS

### Assembly

A total of 24,958,136 paired-end reads (101 bp for each read) were produced for *S. sulcatum* (5,041,543,472 bases, % GC: 52%). After filtering low quality reads, 95.55% reads were retained. As rRNA had been depleted after extraction of the total RNA, it hardly existed in RNA-seq data (0.60%). The remaining rRNA sequences were removed for downstream analyses.

The remaining sequences were assembled with Trinity into 44,633 contigs (Max length: 18,235 bp, Min length: 201 bp, N50: 1498 bp). 94.33% of reads could be properly aligned to contigs in pairs. In order to eliminate assembly errors, 157 *trans* chemiras were detected and removed. Subsequently, the contigs were clustered by high similarity threshold (98%) using CD-HIT and one representative contig for each group was retained (43,437 contigs remained). After linking contigs whose terminals were perfectly matched, the contig number dropped to 43,009. These contigs were then BLASTed to *Oxytricha trifallax* whole genome, and BLAST hit results were contrasted to *O. trifallax* mitochondrial genomic peptides. This revealed that 18 contigs were related to the mitochondrial genome. Given that we used bacteria as the food source during the cultivation of *S. sulcatum*, the assembled contigs probably included a few contaminated sequences from bacteria. Based on BLAST results, we determined that 348 contigs are bacterial. After elimination of the contaminating rDNAs and bacterial sequences, raw reads from *S. sulcatum* were assembled into 42,640 unigenes. Of these unigenes, 27,965 had strong BLAST hits to eukaryotic sequences, and 16,668 had no strong affinity to any domain.

### Gene content in transcriptomes

A variety of methods were used to assess the content of the *Strombidium sulcatum* transcriptome including determining closest relatives for ciliate proteins, identifying most highly expressed genes, CDS region prediction and protein annotation. *Strombidium sulcatum*’s transcriptome had a significant number of BLAST hits to those of *S. inclinatum* and *S. rassoulzadegani* and to the genome of *Oxytricha trifallax*. We identified highly expressed genes as contigs occupping 10% or more of sequence reads with high FPKM (Fragments Per Kilobase of transcript sequence per Millions base pairs sequenced). The functions of about 70% of the contigs with the highest read numbers are unclear. The majority of them have homologous contigs (identity > 90%) in the other 14 ciliates represented in the transcriptome dataset, the exceptions being comp7405_c0 and comp10361_c0, each of which had a blast top hit identity of only about 30%.

After CDS prediction, 40,746 CDS regions were identified from 42,640 unigenes with an average length of 471 aa. At the same time, 40,746 peptide sequences were predicted. With these predicted peptides, we conducted a one-way BLAST search to the GenBank ciliate database against all proteins contained therein; 25,676 of *S. sulcatum* predicted peptides had BLAST results and could be grouped into 16,553 gene groups, 9765 of which were found in the genomes of the other four ciliates (data not shown). The identity distribution of the top hits is shown in Fig. 1. The remaining *S. sulcatum* genes did not satisfy the pairing cut-off criteria (E-value ≤ 1 × 10^−5^).

**Figure 2:**
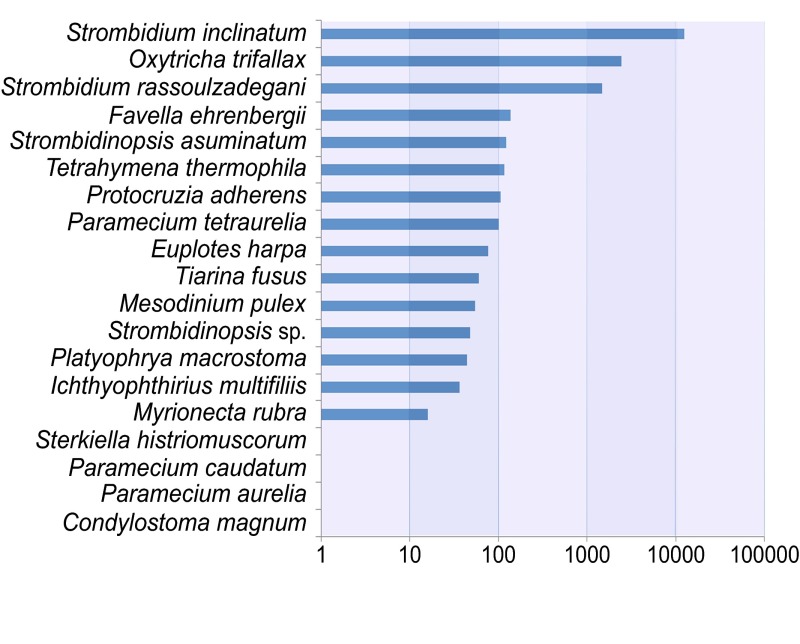
**Species distribution of the most related homologous genes**. The X-axis represents numbers of genes/contigs

Annotation of predicted protein products were matched to models and Swiss-Prot accessions. 20,668 HSPs (Highly Scalable Parallel) were found when blasted to Swiss-Prot database, and each match 16,315 domains in Pfam-A database, 12,201 domains in Superfamily database, 8594 domains in TIGRFAMs database respectively. Assessment of the top BLAST hits’ species distribution for *S. sulcatum* is shown in Fig. 2.

### Ortholog detection and phylogenomics

**Figure 3:**
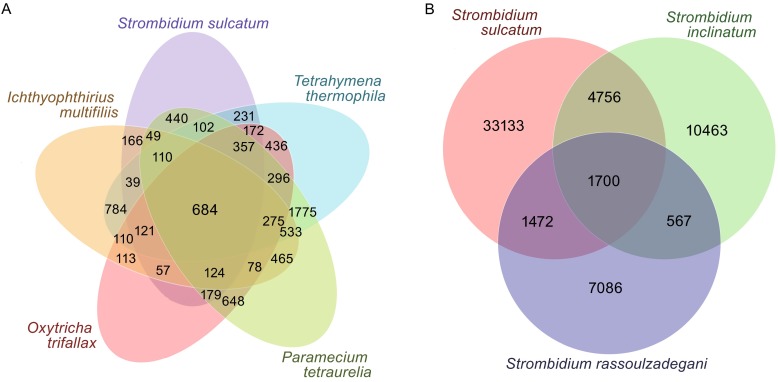
**Comparisons of number of orthologs among** ***Strombidium sulcatum*** **and other ciliates**. Venn diagram showing: (A) shared orthologs among *Strombidium sulcatum* (S) and four other ciliate species for which genomic data are available. Pairwise mutual best-hits by BLASTP were then identified as putative orthologs; (B) orthologs shared among *S. sulcatum*, *S. inclinatum* and *S. rassoulzadegani*. The numbers depict the total number of ortholog groups in each category

A useful approach for surveying the protein-coding gene landscape of a newly sequenced genome is to group genes by orthology, which can provide guidance for functional annotation. Here, we grouped the proteins predicted from *S. sulcatum* transcriptome with four ciliate genomes (*Oxytricha trifallax*, *Tetrahymena thermophila*, *Ichthyophthirius multifiliis* and *Paramecium tetraurelia*) using a reciprocal BLAST hit (RBH) approach. After removing short (<50 aa) and less-conserved (<30% identity) proteins, 684 orthologs were identified as shared by them all. *Strombidium sulcatum* shared most orthologs with *O.* *trifallax*, which is consistent with current systematic arrangements based on morphology and traditional molecular phylogeny (Fig. 3A). In addition, 1700 orthologs were identified in the three *Strombidium* spp. for which transcriptome data are available with *S. sulcatum* sharing most orthologs with *S. inclinatum* (Fig. 3B).

**Figure 4:**
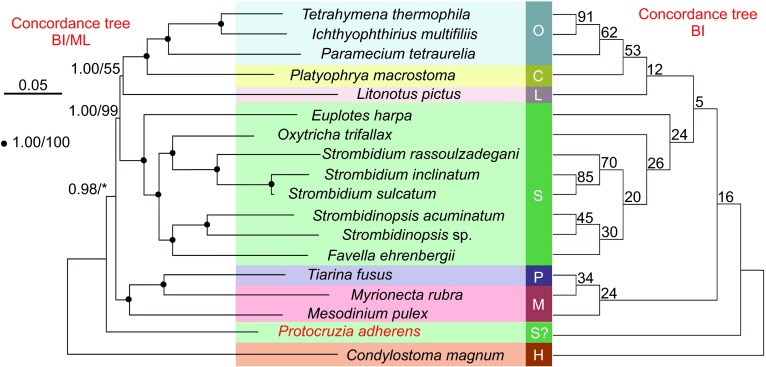
**Comprehensive maximum likelihood phylogenomic tree based on 127 orthologs from 18 ciliates**. Asterisks indicate bootstrap values less than 50% at a given node. The scale bar corresponds to five substitutions per one hundred nucleotide positions in the concatenated tree (left). The numbers depict the concordance factors in the concordance tree (right)

We detected further orthologs among the four ciliate genomes and the 14 ciliate transcriptomes. One hundred and twenty-seven orthologs shared by all 18 ciliates with BLAST identity ≥ 30% and match length ≥ 50 aa were retained to perform phylogenomic analysis. After the 127 ortholog concatenated alignment dataset was produced, and its model was selected as RtREV + I + G + F (gamma shape = 0.947; proportion of invariable sites = 0.063), a concatenated phylogenomic tree was calculated using both maximum likelihood and Bayesian inference methods (Fig. 4). According to the maximum likelihood tree based on 36,724 aa positions from 127 predicted peptide sequences, genera in the class Oligohymenophorea, which includes *Tetrahymena*, *Ichthyophthirius* and *Paramecium*, clustered as a clade with full support. The genus *Platyophrya* (class Colpodea) grouped with the class Oligohymenophorea with full support. The genus *Litonotus* (class Litostomatea) clustered with the class Colpodea and Oligohymenophorea with low support. The ambiguous genus *Protocruzia*, which is known to have an elevated rate of evolution, did not group within any of the major clades. However, most groups with morphological identity are recovered robustly.

The three species of *Strombidium* and *Oxytricha* *trifallax* clustered together with full support, and then grouped with *Euplotes*, *Strombidinopsis* and *Favella*. The *Mesodinium* (class Mesodiniea) did not group together with *Myrionecta* (class Mesodiniea). Instead, *Myrionecta* and *Tiarina* (class Prostomatea) formed a clade with moderate support that was sister to *Mesodinium* (class Mesodiniea) with strong support.

Coalescent analysis was also carried out and a concordance tree was estimated by consolidating all BI trees inferred from the 127 ortholog alignments (Fig. 4). The topology of the concordance tree was similar to that of the concatenated tree. However, *Protocruzia*’s systematic placement was even closer to the class Heterotrichea, while other Spirotrichs clustered as a monophyletic group.

**Figure 5:**
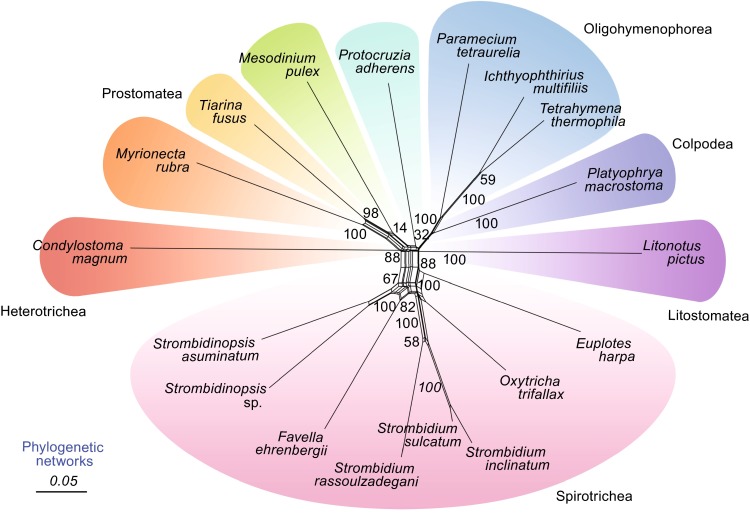
**Phylogenetic network computed from the concatenated orthologs alignment dataset using the neighbornet algorithm and the uncorrected distances**. Numbers along edges are bootstrap support values coming from 1000 replicates. Values <50% are not shown. The scale bar indicates five substitutions per one hundred nucleotide positions

Split decomposition analysis of phylogenetic networks was performed to uncover all possible relationships among the ciliate lineages (Fig. 5). The neighbor-net graphs were calculated based on 36,724 aa from 127 orthologs. The placement of most classes in the networks analysis was generally consistent with both the concatenated and the concordance analyses, and revealed that: (1) *Protocruzia* was in a position that is separate from all existing classes, and; (2) *Myrionecta* was grouped with *Tiarina* which represent the class Prostomatea, as same as the result indicated in maximum likelihood tree.

### Comparative transcriptomic analysis reveals the differential expression of epiplasmic proteins in ciliates

**Figure 6:**
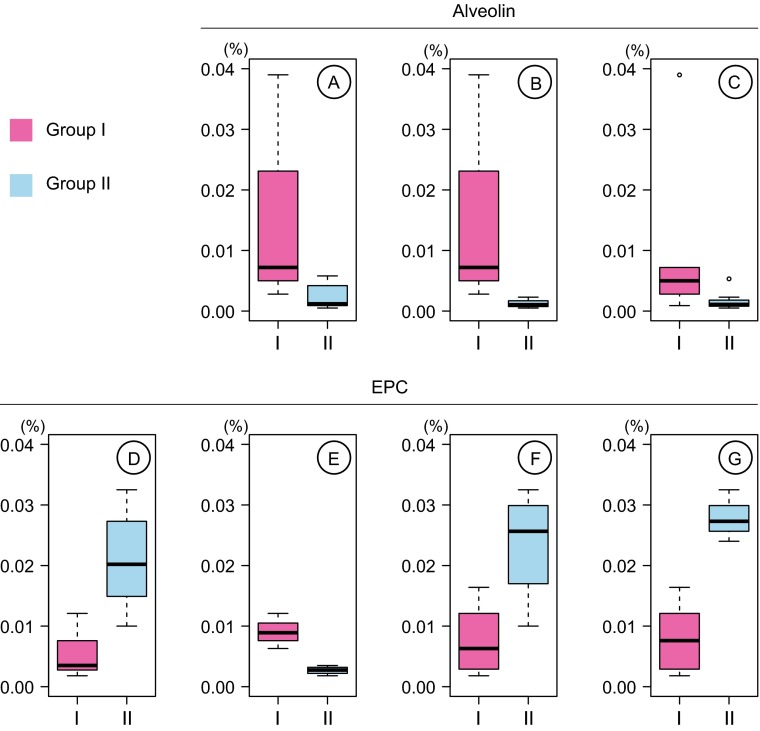
**Proportion of transcripts of the epiplasmin gene family in 13 ciliates divided into groups referring to criteria shown in Table** **1**. The constituents of each group are as follows: (A) criterion 3: Group I [0, 11, 12], Group II [1–10]; (B) criterion 4: Group I [0, 11, 12], Group II [6, 7, 9, 10]; (C) criterion 9: Group I [0, 2–4, 11, 12], Group II [1, 5–10]; (D) criterion 2: Group I [0, 6, 7, 9–12], Group II [1–5, 8]; (E) criterion 4: Group I [0, 11, 12], Group II [6, 7, 9, 10]; (F) criterion 6: Group I [0, 1, 6–12], Group II [2–5]; (G) criterion 11: Group I [0–2, 6–12], Group II [3–5]. Numbers [0–12] in square brackets above are indexes of thirteen ciliates as: 0. *Strombidium sulcatum*; 1. *Platyophrya macrostoma*; 2. *Litonotus pictus*; 3. *Mesodinium pulex*; 4. *Myrionecta rubra*; 5. *Tiarina fusus*; 6. *Euplotes harpa*; 7. *Favella ehrenbergii*; 8. *Protocruzia adherens*; 9. *Strombidinopsis acuminatum*; 10. *Strombidinopsis* sp.; 11. *Strombidium inclinatum*; 12. *Strombidium rassoulzadegani*

*Strombidium* is caharacterized by its pyriform body shape, well-developed adoral zone of membranelles, somatic cilia arranged as a girdle, and the presence of polysaccharide cortical plates in the posterior half of the cell (Fig. 1). In order to reveal the relationship between expression level difference and cellular geometry, morphological information of the thirteen ciliates (*S. sulcatum* plus other 12 ciliates whose RNA-seq transcriptome data were available from MMETSP, *Condylostoma magnum* was not involved because of its inadequate transcriptome data) is summarized in Table 1. Seven potential epiplasmic proteins related to morphology formation were taken into account. Sequences from thirteen of all fourteen species’ transcriptome data (*Condylostoma magnum* was omitted because of the great differences in its transcriptome compared with that of other ciliates) were BLASTed to the epiplasmic proteins dataset (BLASTP, E-value < 1 × 10^−2^, identity > 30%) and homologs were identified in each species. These species, which represented six classes based on morphological characters, were always separated into two groups based on the distinction of one characteristic for each time Table 1. Then, rank-sum tests were performed to both expression level and number of types of epiplasmic protein under several conditions of different group divisions. Some notable results suggest: (1) *Strombidium* spp. have significantly higher expression levels of alveolin than other ciliates (Fig. 6A), including other spirotrichs (Fig. 6B); (2) ciliates that possess extrusomes (e.g. *Strombidium sulcatum*, see (Song et al., 2000) have significantly higher expression levels of alveolin than those that lack extrusomes (Fig. 6C); (3) spirotrichs have significantly lower expression levels of EPC than other ciliates (Fig. 6D), although EPC expression was significantly higher in *Strombidium* spp. than in other spirotrichs (Fig. 6E); (4) ciliates such as spirotrichs with a highly specialized AZM and well-developed cytostome structure, have significantly lower expression levels of EPC than those whose circumoral kinetids and cytostome structure were less well-developed or degenerated (Fig. 6F and 6G). On the other hand, the difference of epiplasmic protein homolog types does not have significant impact on morphological characteristics of these ciliates.

**Table 1:** **Grouping according to each morphological characteristic criterion of 13 ciliate species**

Notes for Table 1. Group 1 members were shaded light orange, Group 2 members were shaded dark orange. (1) All 13 species were divided into 6 groups by classes (criterion 1); (2) all were divided into 2 groups as class Spirotrichea (Group 1) and other classes (Group 2) (criterion 2); (3) all were divided into 2 groups as *Strombidium* spp. (Group 1) and other species (Group 2) (criterion 3); (4) 7 species were divided into 2 groups as *Strombidium* spp. (Group 1) and other species in class Spirotrichea (Group 2) (criterion 4); (5) all 13 species were divided into 2 groups by somatic kineties characteristics, i.e. dikinetid (Group 1) or monokinetid (Group 2) (criterion 5); (6) all were divided into 2 groups by circumoral kineties characteristics, i.e. polykinetids (Group 1) or other types (dikinetid/monokinetid, Group 2) (criterion 6); (7) all were divided into 2 groups by cortical granules characteristics, i.e. cortical granules absent (Group 1) or cortical granules present (Group 2) (criterion 7); (8) all were divided into 2 groups by whether had obvious pellicular alveolus, i.e. not obvious (Group 1) or obvious (Group 2) (criterion 8); (9) all were divided into 2 groups by extrusome characteristics, i.e. extrusomes present (Group 1) or extrusomes absent (Group 2) (criterion 9); (10) all were divided into 2 groups by body size characteristics, i.e. either body length < 100 μm or length-width ratio < 2 (Group 1) or do not accord with that (Group 2) (criterion 10-1 & 10-2); (11) all were divided into 2 groups by buccal area characteristics, i.e. with well-developed buccal structure (Group 1) buccal structure not well-developed (Group 2) (criterion 11)

## DISCUSSION

### Non-model ciliate transcriptome assembly

A problem that has been largely ignored by previous *de novo* transcriptome analyses is the creation of chimeras (Yang & Smith, 2013). Chimeras can come from misassembly of short reads or PCR-induced recombination during library preparation. Chimeras may also be real biological products from gene fusion or transsplicing. Because most chimeras are *trans* chimera in Trinity assembly results, and *cis* chimera detection may lead to false identification according to Yang and Smith (2013), we simply removed all *trans* chimeras.

A second problem in transcriptome assembly of ciliates that cannot be grown axenically is that there may be mitochondrial as well as bacterial transcript-related reads mixed in RNA-seq data. In order to ensure that final unigenes covered only *S. sulcatum* nuclear transcripts, we BLASTed contigs to databases consisting of *Oxytricha trifallax* mitochrondrial genome and bacterial genomes because *Strombidium* is closely related to *Oxytricha*. Although mitochondrial genomes varied significantly among different classes, genes were poorly conserved even among closely related genera, and the gene content and gene order also varied greatly (Swart et al., 2012), only 18 contigs were identified as transcripts related to mitochondria.

### Ortholog detection

The prediction of orthologs and paralogs is a basic step in transcriptome studies, and is usually used to identify the core and auxiliary genes among related organisms (Yang & Smith, 2013). When predicted peptides of four ciliate genomes and the *S. sulcatum* transcriptome are collapsed into ortholog sets, it is found that *S. sulcatum* and *O. trifallax* share more unique orthologs with each other (2005) than are shared between *S. sulcatum* and any of: *T. thermophila* (231), *P. tetraurelia* (440), *I. multifiliis* (166). Within the scope of ciliates, this finding provides direct evidence for the close evolutionary relationship between *S. sulcatum* and *O. trifallax*, and is much stronger than phylogenetic analyses based on sequence data for a single or a few genes.

### Phylogenomic analysis

*Strombidium sulcatum* and two other *Strombidium* spp. clustered strongly in a group nesting deep within the Spirotrichea clade, and were the closest neighbors of *Oxytricha*.

The genus *Protocruzia* did not group within any of the major clades, as reported in a recent work (Gentekaki et al., 2014). The systematic placement of *Protocruzia* has been questioned when it was investigated phylogenetically, especially when based on multi-gene information (e.g. SSU rRNA gene, internal transcribed spacer 2 (ITS2) gene, histone H4 gene), as this usually results in *Protocruzia* being separated from an otherwise monophyletic spirotrichean assembalge (Li et al., 2010). The phylogenomic analyses (both concatenated tree and concordance tree) and phylogenetic network in current study indicated similar findings that *Protocruzia* has a strong impact on the monophyly of the class Spirotrichea, and it might be an early branch of the subphylum Intramacronucleata in the evolutionary line from the class Heterotrichea (subphylum Postciliodesmatophora).

The similarity between the concatenated tree and concordance trees indicates that a robust phylogenetic topological structure could be predicted based on hundreds of molecular loci, even though these had markedly divergent rates of molecular evolution. Furthermore, according to Philippe et al. (2004), missing data in large alignments do not significantly affect inferred phylogeny when resolving relationships among eukaryotic groups. Therefore eukaryotic phylogenomic analysis at the rank of class or higher may be reliable when based on a large data set, even when taxon sampling is limited. This is in contrast to phylogenetic analyses based on a single gene such as the small subunit ribosome DNA (SSU rDNA) which is by far the most commonly used gene marker (Lynn, 2008, Adl et al., 2012). By analysing such a significantly expanded data set, phylogenomics is likely to result in reduced statistical errors and to reveal true evolutionary relationships more reliably.

On the other hand, a tree-like topological structure of relationships among species is assumed in traditional phylogenetic analyses, but recombination, hybridization, gene conversion, and gene transfer can all lead to evolutionary histories that are not adequately modelled by a single tree (Bryant & Moulton, 2004). It is quite likely that binary trees constructed in common analyses failed to reflect the complexity of true evolutionary histories of organisms (Morrison, 2011). Phylogenetic networks can break the restrictive supposition that the topological structure must be tree-like and can be used to visualize genealogical relationships among organisms (Huson et al., 2010). In addition, another advantage of phylogenetic network is to highlight conflicts for different taxa (Hall, 2011).

### Epiplasmic proteins’ transcriptional levels and morphological characteristics of ciliates

As the *Paramecium* epiplasm proteins (Epi1-Epi51) have no counterparts in other organisms (Aubusson-Fleury et al., 2013), we separated the epiplasmins into two types: EpiC (EPC) and Epi1-Epi51 (EPI). So epiplasmic proteins were divided into five classes: (1) the articulins (in various protists) (Marrs & Bouck, 1992, Huttenlauch et al., 1995, Huttenlauch et al., 1998); (2) articulin-like proteins (platein in *Euplotes*) (Kloetzel et al., 2003a); (3) EpiC (EPC, in *Tetrahymena*) (Honts & Williams, 2003); (4) Epi1-Epi51 (EPI, in *Paramecium*) (Damaj et al., 2009, Aubusson-Fleury et al., 2013); and (5) avleolins, perhaps homologues of alveolins and epiplasmins (Gould et al., 2008). Homologs of these five epiplasmic proteins types (articulins, platein, EPC, EPI, avleolins) in thirteen transcriptomes were investigated in this study. In order to test whether these five kinds of epiplasmic proteins’ abundance difference in transcriptional level correlate with present systematic arrangement, we established four grouping criteria (Table 1, criteria 1–4). Rank sum test results suggest transcript abundances of only two epiplasmic protein groups, namely alveolins and EpiC, were significantly correlated to grouping criteria, specifically, criteria 3 (Fig. 6A) & 4 (Fig. 6B) to alveolins and criteria 2 (Fig. 6D) & 4 (Fig. 6E) to EpiC. Interestingly, both of these epiplamic protein groups show a strong correlation in criterion 4, i.e. *Strombidium* versus other species in the class Spirotrichea, which indicates that *Strombidium* spp. have unique cytoskeletal constituents within the class Spirotrichea. On the other hand, homologs of these five epiplasmic proteins do not show much correlation with grouping by present systematic arrangement.

Williams (2004) generated an epiplasm gene *EPC1* knockout construct that was successful in transforming *T. thermophila* cells, and the results suggested that the epiplasm plays a role in the control of either cell shape or cortical development. In this study, preliminary analyses revealed potential correlations between certain morphological characters and differences in the transcriptional levels of epiplasmic proteins. Here we identify seven morphological characteristics as potential grouping criteria for ciliates and which have potential correlations with epiplasmic proteins (Table 1, criteria 5–11). For example, the abundance of transcripts for alveolins is significantly (Sig. = 0.046 < 0.05, criterion 9 in Table 1) correlated with the presence of extrusomes, specifically, the abundance of transcripts for alveolins in species that have extrusomes is higher than that in which lack extrusomes (Fig. 6C). This supports the hypothesis that alveolins are the main constituents of the alveoli, which are part of the cortical epiplasm that includes the extrusomes (Gould et al., 2008). A second example is the correlation between EpiC (EPC) and the presence of circumoral kineties (criterion 6) and the size of the buccal area (criterion 11) (Fig. 6F and 6G). Williams (2004) reported that the most severe abnormality associated with *EPC1* knockout cells was the presence of branched and misaligned membranelles (clusters of cilia) found in the oral apparatus, indicating the importance of this group of epiplasmic proteins in the development of the oral ciliature. The results of the present study of EpiC support these hypotheses. Although present results indicate epiplasmic proteins’ transcriptional levels are potentially correlated with morphological characteristics (such like circumoral kineties, extrusomes and buccal area) of ciliates, further experimental evidence is needed in order to confirm this finding.

## MATERIALS AND METHODS

### Cultures

*Strombidium sulcatum* was grown in 75 cm^2^ plastic culture flasks in filtered marine water with a monoclonal population of *Escherichia coli* as the food source. Cultures were maintained at 25°C for 7–14 days until reaching over 10^6^ cells in total.

### RNA and Illumina Sequencing

Live species were harvested by centrifugation at 1700* g* for 10 min and stored at −80°C until further treatment. The total RNA was extracted using the RNeasy kit (Qiagen, Hilden, Germany) and digested with DNase. The rRNA fraction was depleted using GeneRead rRNA Depletion Kit (Qiagen, Hilden, Germany). The quality of the remaining RNA was assayed using a BioAnalyzer (Agilent Technologies, Palo Alto, CA, USA). In total, 250 ng of the RNA was used to synthesize cDNA using Affymetrix 30 IVT Express Kit (Affymetrix Inc., Santa Clara, CA, USA). The resulting high quality cDNAs were used to construct a library for paired-end sequencing.

### Assembly

The sequencing adapter was trimmed and low quality reads were filtered using FASTX-Toolkit (Gordon & Hannon, 2010). The remaining rRNA reads were removed by mapping to *S. sulcatum*’s 18S rRNAs [GenBank accession: DQ777745] and 5.8S rRNAs [GenBank accession: DQ811089] employing Bowtie v1.0.0 (Langmead et al., 2009). Trinity (r2013-02-25) (Grabherr et al., 2011) was run on a 2.66 GHz, 64 core Intel Xeon 7500 high-performance computing clusters with 512 GB RAM in order to assemble reads from the fastq format paired-end read files from the Illumina Hiseq2000 sequencer. Standard settings for Illumina sequence data and *de novo* transcriptome assembly (with default parameters) were used. After assembly, paired-end reads were aligned to the assembled transcripts for quality assessment using PERL scripts in the trinity package based on Bowtie v1.0.0 (Langmead et al., 2009), following the protocol of Trinity. RNA-seq reads were mapped back to the assembled contigs and were visualized using the inGAP package (Qi et al., 2010, Qi & Zhao, 2011).

The ciliate protein dataset consisted of: (1) peptides of four ciliate genomes (*Ichthyophthirius multifiliis* [Assembly ID: GCA_000220395.1] (Coyne et al., 2011), *Oxytricha trifallax* [Assembly ID: GCA_000295675.1] (Swart et al., 2013), *Paramecium tetraurelia* [Assembly ID: GCA_000165425.1] (Aury et al., 2006), and *Tetrahymena thermophila* [Assembly ID: GCA_000189635.1] (Eisen et al., 2006)) from GenBank; (2) peptides of thirteen transcriptomes (*Platyophrya macrostoma*, *Condylostoma magnum*, *Litonotus pictus*, *Mesodinium pulex*, *Myrionecta rubra*, *Tiarina fusus*, *Euplotes harpa*, *Favella ehrenbergii*, *Protocruzia adherens*, *Strombidinopsis acuminatum*, *Strombidinopsis* sp., *Strombidium inclinatum*, *Strombidium rassoulzadegani*, MMETSP ID see Table S1) from the Marine Microbial Eukaryote Transcriptome Sequencing Project (Resources, 2014); and (3) all ciliate proteins available on GenBank.

*Trans* chimeras were detected and removed following the protocol of Yang and Smith (2013). Assembled sequences were BLASTXed against the ciliate genome/transcriptome protein dataset. Redundancy of contigs was eliminated by CD-HIT v4.6.1 (Fu et al., 2012) (CD-HIT-EST, with 98% sequence identity threshold). Non-redundant contigs were passed to custom PERL scripts that used a BLAST-based strategy to link contigs with others whose terminals were a perfect match (100%). *Oxytricha trifallax* mitochondrial genomic peptides and bacterial genomes database were downloaded from GenBank as a mapping reference to remove reads which were probably contaminated by mitochondria or bacteria.

### Gene prediction and annotation in transcriptomes

Abundance estimation of contigs was analysed using RSEM v1.2.3 (Li & Dewey, 2011) and PERL scripts in the Trinity package based on Bowtie, following the protocol of Trinity. CDS regions were predicted using ESTScan v2.2.1 (Iseli et al., 1999), and only predicted sequences longer than 50 bp were retained. Predicted protein products were blasted to the Swiss-Prot database, and were restricted to the top five HSP bitscores with E-value ≤ 1.0 × 10^−20^. Predicted proteins were matched to HMM models (Pfam-A, Superfamily and TIGRFAMs) using HMMER3 (Eddy, 2009), and were restricted to E-value ≤ 1.0 × 10^−5^ with the top five hits reported.

### Orthologs among *S. sulcatum* and other ciliates

SPOCS (Curtis et al., 2013) (-H -M 2) was employed to identify shared orthologs among *S. sulcatum* and genome peptide data of the other four ciliates. Transcriptome peptide data of 13 ciliates were BLASTed to the *S. sulcatum* predicted peptide dataset by BLASTP (E-value ≤ 1 × 10^−2^, identity ≥ 30%, length ≥ 50 aa), and vice versa. Pairwise mutual best-hits were identified as putative orthologs.

### Phylogenomics

One hundred and twenty-seven orthologs shared by all 18 ciliates were retained and aligned using MUSCLE (Edgar, 2004). Sequence alignments were trimmed by Gblocks v0.91b (Castresana, 2000) (Parameters were set as: Maximum number of contiguous nonconserved positions = 8; Minimum length of a block = 10; Allowed gap positions = with half). A concatenated alignment dataset was produced by putting each ortholog alignment in series using Bioedit v7.2.0 (Hall, 1999). ProtTest v3.3 (Darriba et al., 2011) was employed to select the best model. The Maximum Likelihood (ML) tree was produced by PhyML v3.0 (Guindon et al., 2010). The Bayesian Inference (BI) tree was predicted by MrBayes v3.2.2 (Ronquist & Huelsenbeck, 2003). Coalescent analysis was proformed using Bucky v1.4.2 (Larget et al., 2010) by consolidating 127 BI trees based on 127 ortholog alignments respectively. Clades on concordance trees were annotated with their concordance factor (CF) (Baum, 2007), i.e. the proportion of the genome for which the clade is true. Bayesian concordance analysis was used to obtain point estimates and credibility intervals on CF’s (Ane et al., 2007).

In order to visualize all available phylogenetic signals (36,724 aa in total) in the 127 ortholog alignments, split decomposition analyses were calculated with the computer program SplitsTree ver. 4 (Huson, 1998, Huson & Bryant, 2006). Since we were interested in the ambiguity of relationships of taxa at class level, phylogenetic networks were generated for the concatenated dataset of 127 ortholog alignments using the neighbornet algorithm with uncorrected distances (Bryant & Moulton, 2004). To assess the reliability of the phylogenetic networks, bootstrap analyses with 1000 replicates were carried out.

### Epiplasmic protein expression analyses based on thirteen ciliates’ transcriptome data

Sequences of the major epiplasmic proteins (EPI, Articulin, EPC, Platein, Alveolin) were acquired from GenBank and ParameciumDB (http://paramecium.cgm.cnrs-gif.fr/) and formed the epiplasmic proteins dataset (Acc. No. see Table S2). This epiplasmic proteins dataset were used as queries to BLASTed (E-value ≤ 1.0 × 10^−2^, identity ≥ 30%) aganist the transcriptome data of these ciliate species in order to find homologs of epiplasmic proteins. Relative transcript abundance (number of reads mapping to the gene/total number of all reads of this species) and homolog type numbers of these genes were acquired from their transcriptome mapping data (Resources, 2014).

Morphologically similar groups were determined based on four taxonomic arrangements (different classes; the class Spirotrichea versus other classes; *Strombidium* spp. versus other ciliates; *Strombidium* spp. versus other species in the class Spirotrichea) and seven morphological character states concerning: somatic kineties, circumoral kineties, cortical granules, pellicular alveolus, extrusomes, cell size and shape, and the buccal area (Agatha, 2004, Foissner et al., 2007). Morphological character states were collected from morphological descriptions of each species as follows: *Strombidium sulcatum* Claparède & Lachmann, 1858 (Song et al., 2000), *Strombidium inclinatum* Montagnes, 1990 (Montagnes et al., 1990), *Strombidium rassoulzadegani* McManus, 2010 (McManus et al., 2010), *Favella ehrenbergii* (Claparède and Lachmann, 1858) Jörgensen, 1924 (Kim et al., 2010), *Strombidinopsis acuminatum* Faure-Fremiet, 1924 (Lynn et al., 1991, Dale & Lynn, 1998, Kim et al., 2010), *Euplotes harpa* Stein, 1859 (Dragesco et al., 1986), *Litonotus pictus* Gruber, 1884 (Kim & Min, 2009), *Platyophrya macrostoma* Foissner, 1980 (Foissner, 1993), *Protocruzia adherens* (Mansfeld, 1923) Kahl, 1930 (Song & Wilbert, 1997), *Mesodinium pulex* Claparède & Lachmann, 1858 (Tamar, 1992), *Myrionecta rubra* (Lohmann 1908) Jankowski, 1976 (Lynn, 2008) and *Tiarina fusus* (Claparède and Lachmann, 1859) Bergh, 1882 (Corliss, 1979). SPSS v16.0 (Inc, 2007) was employed to perform rank sum test among groups.

## Electronic supplementary material

Below is the link to the electronic supplementary material.

Table S1. RNA-seq dataset information of thirteen ciliates used in this work. Supplementary material 1 (XLSX 10 kb)

Table S2. List of major epiplasmic proteins in ciliates.Supplementary material 2 (DOC 82 kb)
