# Supplementary material for: Phylogenomics of non-model ciliates based on transcriptomic analyses
Source: Protein Cell. 2015 Apr 2;6(5):373–85. doi: 10.1007/s13238-015-0147-3 (PMC4417680; doi:10.1007/s13238-015-0147-3)
Supplement: Supplementary file 2 — Table S2. List of major epiplasmic proteins in ciliates.Supplementary material 2 (DOC 82 kb) [file 13238_2015_147_MOESM2_ESM.doc]

**Table S2.** List of major epiplasmic proteins in ciliates.

| Database | Acc. No. | Gene/Protein | Species |
| --- | --- | --- | --- |
| GenBank | XP_627385 | Articulin family protein | *Cryptosporidium parvum Iowa II* |
| GenBank | XP_626594 | Articulin family protein | *Cryptosporidium parvum Iowa II* |
| GenBank | XP_625909 | Articulin family protein | *Cryptosporidium parvum Iowa II* |
| GenBank | XP_625908 | Articulin family protein | *Cryptosporidium parvum Iowa II* |
| GenBank | XP_625571 | Articulin family protein | *Cryptosporidium parvum Iowa II* |
| GenBank | XP_626020 | Articulin family protein | *Cryptosporidium parvum Iowa II* |
| GenBank | CDJ68256 | Articulin family protein | *Eimeria necatrix* |
| GenBank | AAB23241 | Articulin | *Euglena gracilis* |
| GenBank | AAB23240 | Articulin | *Euglena gracilis* |
| GenBank | AAM94464 | Beta-/gamma-platein precursor | *Euplotes aediculatus* |
| GenBank | AAM94463 | Alpha-2 platein precursor | *Euplotes aediculatus* |
| GenBank | AAM94462 | Alpha-1 platein precursor | *Euplotes aediculatus* |
| GenBank | XP_003884417 | Articulin family protein, related | *Neospora caninum Liverpool* |
| GenBank | XP_003881475 | Articulin family protein, related | *Neospora caninum Liverpool* |
| GenBank | XP_003880121 | Putative articulin 4 | *Neospora caninum Liverpool* |
| ParameciumDB | GSPATG00021018001 | EPI1 | *Paramecium tetraurelia* |
| ParameciumDB | GSPATG00022926001 | EPI2 | *Paramecium tetraurelia* |
| ParameciumDB | GSPATG00018381001 | EPI3 | *Paramecium tetraurelia* |
| ParameciumDB | GSPATG00018839001 | EPI4 | *Paramecium tetraurelia* |
| ParameciumDB | GSPATG00010399001 | EPI5 | *Paramecium tetraurelia* |
| ParameciumDB | GSPATG00037619001 | EPI6 | *Paramecium tetraurelia* |
| ParameciumDB | GSPATG00033449001 | EPI7 | *Paramecium tetraurelia* |
| ParameciumDB | GSPATG00008953001 | EPI8 | *Paramecium tetraurelia* |
| ParameciumDB | GSPATG00025293001 | EPI9 | *Paramecium tetraurelia* |
| ParameciumDB | GSPATG00000592001 | EPI10 | *Paramecium tetraurelia* |
| ParameciumDB | GSPATG00032435001 | EPI11 | *Paramecium tetraurelia* |
| ParameciumDB | GSPATG00015528001 | EPI12 | *Paramecium tetraurelia* |
| ParameciumDB | GSPATG00012091001 | EPI13 | *Paramecium tetraurelia* |
| ParameciumDB | GSPATG00009363001 | EPI14 | *Paramecium tetraurelia* |
| ParameciumDB | GSPATG00019848001 | EPI15 | *Paramecium tetraurelia* |
| ParameciumDB | GSPATG00007885001 | EPI16 | *Paramecium tetraurelia* |
| ParameciumDB | GSPATG00005772001 | EPI17 | *Paramecium tetraurelia* |
| ParameciumDB | GSPATG00024301001 | EPI18 | *Paramecium tetraurelia* |
| ParameciumDB | GSPATG00008095001 | EPI19 | *Paramecium tetraurelia* |
| ParameciumDB | GSPATG00011787001 | EPI20 | *Paramecium tetraurelia* |
| ParameciumDB | GSPATG00005649001 | EPI21 | *Paramecium tetraurelia* |
| ParameciumDB | GSPATG00026586001 | EPI22 | *Paramecium tetraurelia* |
| ParameciumDB | GSPATG00023977001 | EPI23 | *Paramecium tetraurelia* |
| ParameciumDB | GSPATG00012673001 | EPI24 | *Paramecium tetraurelia* |
| ParameciumDB | GSPATG00013326001 | EPI25 | *Paramecium tetraurelia* |
| ParameciumDB | GSPATG00006591001 | EPI26 | *Paramecium tetraurelia* |
| ParameciumDB | GSPATG00002128001 | EPI27 | *Paramecium tetraurelia* |
| ParameciumDB | GSPATG00013569001 | EPI28 | *Paramecium tetraurelia* |
| ParameciumDB | GSPATG00016851001 | EPI29 | *Paramecium tetraurelia* |
| ParameciumDB | GSPATG00001502001 | EPI30 | *Paramecium tetraurelia* |
| ParameciumDB | GSPATG00002556001 | EPI31 | *Paramecium tetraurelia* |
| ParameciumDB | GSPATG00025640001 | EPI32 | *Paramecium tetraurelia* |
| ParameciumDB | GSPATG00030876001 | EPI33 | *Paramecium tetraurelia* |
| ParameciumDB | GSPATG00017440001 | EPI34 | *Paramecium tetraurelia* |
| ParameciumDB | GSPATG00019287001 | EPI35 | *Paramecium tetraurelia* |
| ParameciumDB | GSPATG00007006001 | EPI36 | *Paramecium tetraurelia* |
| ParameciumDB | GSPATG00016662001 | EPI37 | *Paramecium tetraurelia* |
| ParameciumDB | GSPATG00009618001 | EPI38 | *Paramecium tetraurelia* |
| ParameciumDB | GSPATG00021998001 | EPI39 | *Paramecium tetraurelia* |
| ParameciumDB | GSPATG00035145001 | EPI40 | *Paramecium tetraurelia* |
| ParameciumDB | GSPATG00025744001 | EPI41 | *Paramecium tetraurelia* |
| ParameciumDB | GSPATP00038597001 | EPI42 | *Paramecium tetraurelia* |
| ParameciumDB | GSPATP00017377001 | EPI43 | *Paramecium tetraurelia* |
| ParameciumDB | GSPATP00019341001 | EPI44 | *Paramecium tetraurelia* |
| ParameciumDB | GSPATP00007177001 | EPI45 | *Paramecium tetraurelia* |
| ParameciumDB | GSPATP00010287001 | EPI46 | *Paramecium tetraurelia* |
| ParameciumDB | GSPATP00017294001 | EPI47 | *Paramecium tetraurelia* |
| ParameciumDB | GSPATP00018713001 | EPI48 | *Paramecium tetraurelia* |
| ParameciumDB | GSPATP00027525001 | EPI49 | *Paramecium tetraurelia* |
| ParameciumDB | GSPATP00032507001 | EPI50 | *Paramecium tetraurelia* |
| ParameciumDB | GSPATP00033927001 | EPI51 | *Paramecium tetraurelia* |
| GenBank | AAC15831 | Articulin 4 | *Pseudomicrothorax dubius* |
| GenBank | AAC15830 | Articulin 1 | *Pseudomicrothorax dubius* |
| GenBank | AAB02426 | Articulin p60 | *Pseudomicrothorax dubius* |
| GenBank | AAF85984 | Epiplasmin C | *Tetrahymena pyriformis* |
| GenBank | AAM44065 | Epiplasmin C | *Tetrahymena thermophila* |
| GenBank | ACO05021 | Alveolin 2 | *Tetrahymena thermophila* |
| GenBank | XP_002370041 | Articulin 4 | *Toxoplasma gondii ME49* |
